# Effectiveness of Digital Health Interventions Containing Game Components for the Self-management of Type 2 Diabetes: Systematic Review

**DOI:** 10.2196/44132

**Published:** 2023-06-01

**Authors:** Linda Ossenbrink, Tina Haase, Patrick Timpel, Olaf Schoffer, Madlen Scheibe, Jochen Schmitt, Stefanie Deckert, Lorenz Harst

**Affiliations:** 1 Center for Evidence-based Healthcare University Hospital and Faculty of Medicine Carl Gustav Carus Technische Universität Dresden Dresden Germany

**Keywords:** diabetes, gamification, digital health, diabetes self-management, mobile phone

## Abstract

**Background:**

Games and game components have become a major trend in the realm of digital health research and practice as they are assumed to foster behavior change and thereby improve patient-reported and clinical outcomes for patients with type 2 diabetes.

**Objective:**

The aim of this systematic review was to summarize and evaluate the current evidence on the effectiveness of digital health interventions containing game components on behavioral, patient-reported, and clinical outcomes for patients with type 2 diabetes.

**Methods:**

An electronic search was conducted in MEDLINE and PsycINFO in April 2020; updated in April 2022; and supplemented by additional searches via Google Scholar, Web of Science (which was used for forward citation tracking), and within the references of the included records. Articles were identified using predefined inclusion and exclusion criteria. In total, 2 reviewers independently conducted title, abstract, and full-text screening and then individually performed a critical appraisal of all the included studies using the Cochrane risk-of-bias tool version 2. A consensus was reached through discussion.

**Results:**

Of 2325 potentially relevant titles (duplicates excluded), 10 (0.43%) randomized controlled trials were included in this review. Quality assessment revealed a high risk of bias for all randomized controlled trials except for 10% (1/10), with performance bias due to the lack of blinding being the major source of bias. There is evidence suggesting that digital health interventions containing game components can substantially improve motivation for physical activity (1/1, 100% of the studies dealing with PA motivation), exercise intensity (3/5, 60%), dietary behavior (4/4, 100%), health literacy (1/3, 33%), mental quality of life (2/2, 100%), glycated hemoglobin level (2/6, 33%), BMI (1/3, 33%), fasting plasma glucose level (1/2, 50%), waist circumference (1/1, 100%), and aerobic capacity (1/1, 100%).

**Conclusions:**

Published studies indicated that digital health interventions containing game components might improve health behavior patterns, quality of life, and clinical outcomes in patients with type 2 diabetes. However, the intervention types and outcomes studied were heterogeneous, and study quality was mostly low, which translates to ambiguous results. Future research should focus on sound methodology and reporting as well as on identifying game components that contribute to significant positive effects.

**Trial Registration:**

PROSPERO CRD42020209706; https://www.crd.york.ac.uk/prospero/display_record.php?RecordID=209706

## Introduction

### Background

According to the 2021 and most recent Diabetes Atlas provided by the International Diabetes Federation, diabetes affects 537 million people worldwide, which equals a share of almost 7% of the world’s total population [1]. Compared with the data provided in the 2019 Atlas, this is an increase in diabetes prevalence of almost 16% within 2 years [1]. The vast majority of people diagnosed with diabetes (95%) live with type 2 diabetes [2,3].

Apart from genetic predispositions and a higher probability of having a diabetes diagnosis at an older age, several risk factors of type 2 diabetes are lifestyle-related, such as physical inactivity; malnutrition; and, correspondingly, overweight and obesity [4]. Therefore, several clinical practice guidelines on the treatment of type 2 diabetes focus not only on pharmacological interventions but also on a comprehensive self-management regimen that includes theory-based behavior change [4-7]. Measures for the latter warranted by the so-called Diabetes Self-Management and Education (DSME) regimen defined by the American Diabetes Association (ADA) include education on symptoms, etiology of and coping with diabetes, the adoption of a healthy (ie, high fiber- and fruit- and vegetable-based) diet, and the uptake of regular physical activity (PA) as a means to achieve weight loss [4,6]. Apart from the education component, continuous monitoring of blood glucose values, food intake, and frequency and intensity of PA is a requirement of DSME [6].

The potential of digital health applications, such as telemedicine, for supporting patients regarding DSME is well documented for patients with a more recent diabetes diagnosis, especially for applications that enable continuous glucose self-monitoring or health care provider feedback on the values recorded [8].

Recently, interest has spiked in digital health applications containing game components as they are expected to offer aid in behavior change [9], which, for many patients, is a necessary precondition for successful DSME [10]. Game components such as scoring systems, trophies, and leaderboards have been shown to be effective in increasing the motivation for uptake of healthy behaviors in a number of chronic conditions [11] as well as a measure of health promotion. However, the methodological quality of the evidence is still moderate to low [12]. Educational games often rely on storytelling elements such as coherent narratives and episodes, which generate a so-called transportation effect where players immerse themselves completely in the narrative world, which loosens reluctance toward behaviors perceived as laborious or unpleasant, such as PA [13,14]. Exergames stimulate PA by challenging the players’ abilities and rewarding success [15].

A moderating role of the regulatory mode can be assumed in the relationship between gaming and behavior change. According to theory, either individuals can assess the situation they are in and then develop the most adequate strategy to reach a behavioral aim (assessment) or they can just initiate the behavior for which they strive (locomotion). Locomotion is associated with higher intrinsic motivation, whereas assessment is associated with anticipating failure and, therefore, procrastination [16].

In 2016, a meta-analysis including both types of game-based interventions (educational games and exergames) for patients with diabetes (type 1 and 2) showed no effect on blood glucose values (glycated hemoglobin [HbA_1c_]) but showed an effect on quality of life, balance, and muscle strength [17]. Theng et al [18] found that videogames were useful tools for diabetes education independent of diabetes type. However, the analyses are outdated (both searches were conducted in 2014) given the substantial increase in the availability of gamified health interventions, as evidenced by a recent overview of gamified interventions used in the context of diabetes [19], half of which were launched after 2016. The median survival time of reviews is 5.5 years before they are outdated [20]. In addition, patient populations and the measures taken to deal with type 1 and type 2 diabetes differ greatly [7], and so do the effective components of digital health applications [8]. Therefore, targeted game-based interventions are necessary as well. Furthermore, given the complex requirements of DSME, focusing the analysis solely on clinical outcomes and neglecting behavioral outcomes as well as patient-reported outcomes (PROs), as did Martos-Cabrera et al [21] and Kaihara et al [22], is limited in perspective.

### Objectives

Therefore, the questions to be answered were as follows: (1) *Do digital game-based interventions have an effect on the health behavior of patients with type 2 diabetes?* and (2) *Do digital game-based interventions have an effect on clinical outcomes and PROs in patients with type 2 diabetes?*

## Methods

The protocol for this systematic review was published beforehand in PROSPERO (CRD42020209706) and followed during the conduct of the review. Reporting of the results adhered to the PRISMA (Preferred Reporting Items for Systematic Reviews and Meta-Analyses) statement [23].

### Study Inclusion and Exclusion

The inclusion and exclusion criteria were defined according to the population, intervention, control, outcome, and study design scheme (Textboxes 1 and 2). For this purpose, digital health interventions containing game components were defined as the intervention group, whereas usual care or the use of digital health interventions without a game component were defined as the control group.

**Study inclusion criteria.**

**Population**

- Participants with type 2 diabetes (no age restriction)

**Intervention**

- Use of digital health applications containing game components identified in previous reviews [12,17] (such as virtual reality, serious gaming, or exergaming)

**Comparison**

- Use of digital health applications without game components or standard or usual care

**Outcome**

- Primary outcomes:
  Behavioral outcomes such as physical activity or dietary behavior
- Secondary outcomes:
  Patient-reported outcomes such as self-efficacy, patient empowerment, and quality of life
  Clinical parameters such as glycated hemoglobin (blood sugar value), BMI, and systolic blood pressure or diastolic blood pressure

**Study design**

- Randomized controlled trials
- Nonrandomized studies (only when n>10)

**Study exclusion criteria.**

**Population**

- Participants without diabetes or with type 1 or gestational diabetes

**Intervention**

- No treatment or intervention

**Comparison**

- No treatment or intervention

**Outcome**

- Neither behavioral outcomes nor patient-reported outcomes or clinical parameters studied

**Study design**

- Cross-sectional studies, qualitative studies, reviews, and meta-analyses

Only studies published in English or German were included.

### Database Search

An electronic database search was conducted in MEDLINE (via PubMed) and PsycINFO to cover both medical and psychological research. The initial search was conducted in April 2020 and updated in April 2022 with no restrictions on start time. The search string (Multimedia Appendix 1) included terms covering type 2 diabetes (population), including Medical Subject Heading terms and synonyms for *game* and *gaming* (intervention) as well as game components, and was piloted in previous research [24]. Population and intervention terms were linked with the operator *AND*. No restrictions were imposed on the outcome category of the search string to avoid accidentally excluding relevant effects of gamified interventions.

Additional searches were conducted within the references of the included studies (backward citation tracking) as well as on Google Scholar and Web of Science, where publications citing the included studies were checked (forward citation tracking) in July 2022.

### Screening and Data Extraction

The screening of relevant records was a 2-step process. First, 2 independent reviewers screened the titles and abstracts of all records found by the database and hand searches. If deemed relevant by at least one reviewer, the full text was assessed for eligibility by both reviewers as well. The reference manager EndNote (Clarivate Analytics) was used for both screening and duplicate removal.

Data were extracted according to the population, intervention, control, outcome, and study design scheme, aiming at allowing for a comparison of the effects of different gamified intervention types on the aforementioned outcome domains. In addition, the following information was extracted from each study: (1) bibliographic information, (2) population characteristics, (3) the allocation of the study participants to the intervention and control group or control groups, (4) treatment or interventions applied to the control group (as a means to account for plausible confounding factors), (5) inclusion and exclusion criteria of each applicable study (as a means to account for plausible confounding factors), and (6) outcome measures (to inform quality assessment).

The data extraction sheet was piloted by 2 researchers on 2 of the included studies, which were randomly selected, and subsequently slightly adjusted by including the category “outcome measures.” Data extraction was performed using a Microsoft Excel (Microsoft Corp) spreadsheet.

For a visual representation, HbA_1c_ values at baseline and after intervention completion were extracted in percentage or mmol/mol depending on the data available in the included studies, along with the SD. Δ HbA_1c_ was computed, and statistical significance was extracted from the included studies. The threshold for statistically significant effects was set at *P*<.05. No assumptions were made if information was missing; this was labeled as “not reported” instead. All study results were tabulated. Apart from the tabulation and visualization of HbA_1c_ results, the presentation of the results is narrative.

### Quality Assessment

The randomized controlled trials (RCTs) found were assessed for study quality by applying the Cochrane risk-of-bias tool version 2 (RoB 2) [25], whereas cohort studies, if included, were assessed using the corresponding Critical Appraisal Skills Programme (CASP) cohort study checklist [26]. Case-control studies were assessed using the corresponding checklist also provided by the CASP [27]. The RoB 2 covers bias within an outcome resulting from the randomization process (selection bias); blinding of participants, assessors, and analysts; deviations from intervention delivery (performance bias); changes in participants’ adherence to the intervention (attrition bias); modalities of outcome measurement; or selective reporting (reporting bias). RoB 2 assessment was performed for each relevant study outcome according to our inclusion criteria. According to the RoB 2 manual, the overall risk of bias within a study was deemed high if 1 study outcome had a high risk of bias. Both records by Höchsmann et al [28,29] were treated as 1 study with several outcomes. CASP checklists for nonrandomized studies cover the same categories except for randomization and blinding while also putting an emphasis on practical implications of the study results. In contrast to the RoB 2, CASP checklists were applied to the entire study instead of to selected outcomes. The RoB 2 deems studies to be at a low risk of bias when a low risk of bias is detected for all relevant domains. Some concerns regarding the risk of bias within an outcome can be assumed when some concerns are raised for at least one domain. A high risk of bias can be assumed when multiple domains raise some concerns or a high risk of bias is detected for at least one domain [25]. With the CASP, overall risk of bias is assessed by answering the following question: “Do you believe the results?”

Quality assessment was performed for the effect of both assignment to the intervention (ie, the intention-to-treat effect) and adherence to the intervention (ie, the per-protocol effect).

All steps of the review—screening of titles and abstracts and full texts, data extraction, and quality assessment—were performed by at least 2 researchers independently (LO, LH, or PT in the first search period and LH and TH in the second search period) to minimize bias. Differences in inclusion, extraction, and quality assessment were resolved through discussion with a third person not involved in the screening process (SD).

### Diversions From the Protocol

Contrary to the protocol registered with PROSPERO, research question 2 was adapted so that it clearly addressed PROs. Furthermore, we refrained from performing a search in key journals as these were all listed in MEDLINE.

## Results

### Results of the Search Process

**Figure 1:**
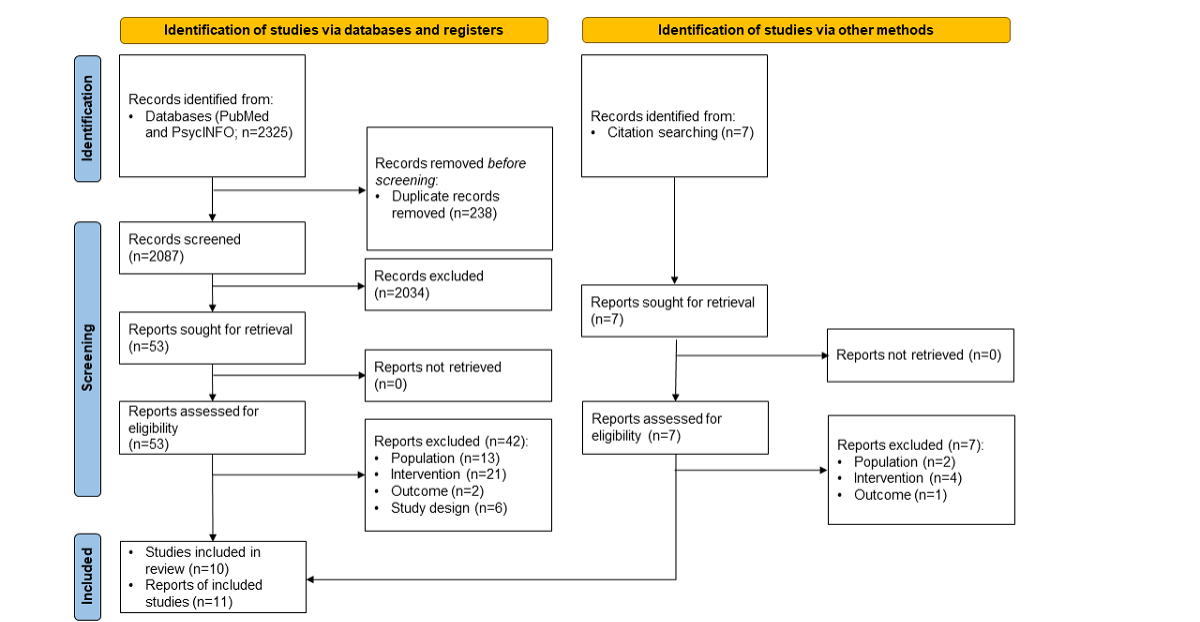
PRISMA (Preferred Reporting Items for Systematic Reviews and Meta-Analyses) flow diagram depicting study inclusion.

Both database searches (2020 and 2022) taken together yielded 2325 results. A total of 7 additional potentially relevant publications were identified via additional searches, but excluded after full text assessment. In total, 2087 publications remained after duplicates were removed, 2034 (97.5%) of which were removed after title and abstract screening. The full texts of the remaining 53 publications were assessed for eligibility. This process led to the exclusion of 25% (13/53) of the studies as they addressed a population outside this review’s scope, such as patients with type 1 or prediabetes [30-42]. Another 40% (21/53) of the studies were excluded as the interventions studied did not have a game component [43-63]. A total of 15% (8/53) of the studies were excluded as they used 1-armed designs or did not study any of the prespecified outcomes but solely patient experiences, such as satisfaction with the application [64-71]. A complete list of the excluded studies with reasons for exclusion can be found in Multimedia Appendix 2 [30-71]. Finally, 10 studies were included in the qualitative data analysis [28,29,72-80]. Höchsmann et al [28,29] reported results from the same study in 2 records. The process of study selection is depicted in Figure 1.

### Descriptive Statistics of the Populations Studied

All the included studies (10/10, 100%) were RCTs. However, Kempf and Martin [78] and Brinkmann et al [72] applied a crossover design in which participants in the control group received the intervention later during the study period. The study samples ranged from 8 participants [72] to 465 participants [76]. The lowest mean age was 44 (SD 7.9) years [79] and the highest mean age was 68 (SD 5.8) years [73]. Brinkmann et al [72] only reported the age span of the study participants, which was 67 to 75 years. Overall, 533 female participants and 1061 male participants were included. The intervention duration ranged from 30 minutes (exergame) [72] to 36 weeks (self-management app with quiz component) [74]. In total, 30% (3/10) of the RCTs performed follow-up analyses after the intervention period, with follow-up times ranging from 7 days [72] to 48 weeks [77]. A complete overview of the population characteristics of the included RCTs can be found in Table 1.

**Table 1:** Study and population characteristics of the included randomized controlled trials.

Study, year, title, journal, and country	Study design	Study duration	Follow-up time	Population characteristics				
				Sample size, n	Age (years)	Sex	Inclusion criteria	Exclusion criteria

Brinkmann et al [72], 2017, “Effects of Cycling and Exergaming on Neurotrophic Factors in Elderly Type 2 Diabetic Men—A Preliminary Investigation”/Experimental and Clinical Endocrinology & DiabetesExp Clin Endocrinol Diabetes, Germany	Individually randomized crossover trial	30 min once	None	8	Ranging from 67 to 75	8 male participants	Not reported	Nonsmoking Absence of diabetic retinopathy, neuropathy, nephropathy, or cardiovascular complications
Dugas et al [73], 2018, “Individual Differences in Regulatory Mode Moderate the Effectiveness of a Pilot mHealth trial for Diabetes Management among older Veterans”/PLOS ONE^a^, United States	Individually randomized controlled trial	13 weeks	None	27	Mean 67.8 (SD 6.1)	Not reported	Veteran patients with type 2 diabetes Affiliated with a Veterans Affairs medical center Aged >60 years Poorly controlled diabetes (HbA_1c_^b^ >7.9%)	Blindness Deafness Serious mental illness Homelessness
Glasgow et al [74], 2010, “Outcomes of Minimal and Moderate Support Versions of an Internet-Based Diabetes Self-Management Support Program”/Journal of General Internal Medicine, United States	Individually randomized controlled trial	16 weeks	None	463	Mean 58.4 (SD 9.2)	233 male and 230 female participants	Aged 25 to 75 years Type 2 diabetes diagnosis BMI >25 kg/m² At least one or more risk factor for CVD^c^ Telephone access Internet access at least twice a week	None
Grewal et al [75], 2015, “Sensor-Based Interactive Balance Training with Visual Joint Movement Feedback for Improving Postural Stability in Diabetics with Peripheral Neuropathy: A Randomized Controlled Trial”/Gerontology, United States	Individually randomized controlled trial	45 min twice a week for 4 weeks	None	39	Mean 63.7 (SD 8.2)	20 male and 19 female participants	Ability to walk on one’s own for 20 meters Type 2 diabetes Peripheral neuropathy	Diagnosis of cognitive, vestibular, or central neurological dysfunction Diagnosis of musculoskeletal abnormality Active foot ulcers Charcot joints History of balance disorder
Höchsmann et al [28,29], 2019, “Effectiveness of a Behavior Change Technique–Based Smartphone Game to Improve Intrinsic Motivation and Physical Activity Adherence in Patients With Type 2 Diabetes: Randomized Controlled Trial”/JMIR Serious Games and “Novel Smartphone Game Improves Physical Activity Behavior in Type 2 Diabetes”/American Journal of Preventive Medicine, Switzerland	Individually randomized controlled trial	24 weeks	None	36	Mean 57 (SD 5.5)	19 male and 17 female participants	Physically inactive (<150 min of moderate-intensity PA^d^ per week) BMI >25 kg/m² Type 2 diabetes Non–insulin-dependent Aged 45 to 70 years Having used a smartphone regularly for 1 year before the study	Health risks counterindicating PA Impaired mobility Acute infections Injuries
Kempf and Martin [78], 2013, “Autonomous Exercise Game Use Improves Metabolic Control and Quality of Life in Type 2 Diabetes Patients—a Randomized Controlled Trial”/BMC Endocrine Disorders, Germany	Crossover individually randomized controlled trial	30 min per day for 12 weeks	None	220	Mean 62 (SD 11; [intervention group]) and 60 (SD 9; [control group])	119 female and 101 male participants	Type 2 diabetes Diagnosis>5 years ago Aged 50 to 75 years BMI >27 kg/m² Included in disease management program for diabetes	Regular PA Pharmacological therapy (except metformin and DPP-4^e^ inhibitors)
Kerfoot et al [76], 2017, “A Team-Based Online Game Improves Blood Glucose Control in Veterans With Type 2 Diabetes: A Randomized Controlled Trial”/Diabetes Care, United States	Individually randomized controlled trial	24 weeks	48 weeks	465	Mean 59.5 (SD 9.9)	28 female and 428 male participants	Type 2 diabetes Inadequate glucose control Taking oral diabetes medication Insulin- and non–insulin-dependent	Not reported
Koohmareh et al [79], 2020, “Effect of Implementing a Mobile Game on Improving Dietary Information in Diabetic Patients”/Medical Journal of The Islamic Republic of Iran, Iran	Individually randomized controlled trial	15 min per day for 6 weeks	None	60	Mean 44.1 (SD 7.9; control group) and 43.9 (SD 9.0; intervention group)	32 female and 28 male participants	Type 2 diabetes diagnosis confirmed by a specialist Aged >18 years Minimal literacy Android-run smartphone Smartphone skills Willingness to participate	Not reported
Maharaj et al [80], 2021, “Comparing Two Commercially Available Diabetes Apps to Explore Challenges in User Engagement: Randomized Controlled Feasibility Study”/JMIR Formative Research, Australia and New Zealand	Individually randomized controlled trial	2 weeks	None	89	Mean 53.2 (SD 11.1; control group) and 52.6 (SD 13.0; intervention group)	31 female and 58 male participants	Type 2 diabetes diagnosis confirmed by a specialist Aged >18 years Fluent in spoken and written English iOS- or Android-run smartphone Written consent	Not reported
Turnin et al [77], 2021, “Impact of a Remote Monitoring Programme Including Lifestyle Education Software in Type 2 Diabetes: Results of the Educ@dom Randomised Multicentre Study”/Diabetes Therapy, France	Individually randomized controlled multicenter study	48 weeks	None	263	Mean 59.6 (SD 9.6)	57 female and 166 male participants	Type 2 diabetes diagnosis confirmed by a specialist Aged >18 years Insulin- and non–insulin-dependent HbA_1c_ of 6.5% to ≤10% Active internet connection	Active severe comorbidities Reduced mobility Eating disorders Bariatric surgery

^a^PLOS ONE: Public Library of Science ONE.

^b^HbA_1c_: glycated hemoglobin (blood sugar value).

^c^CVD: cardiovascular disease.

^d^PA: physical activity.

^e^DPP-4: dipeptidyl peptidase 4

### Results of the Quality Assessment

**Figure 2:**
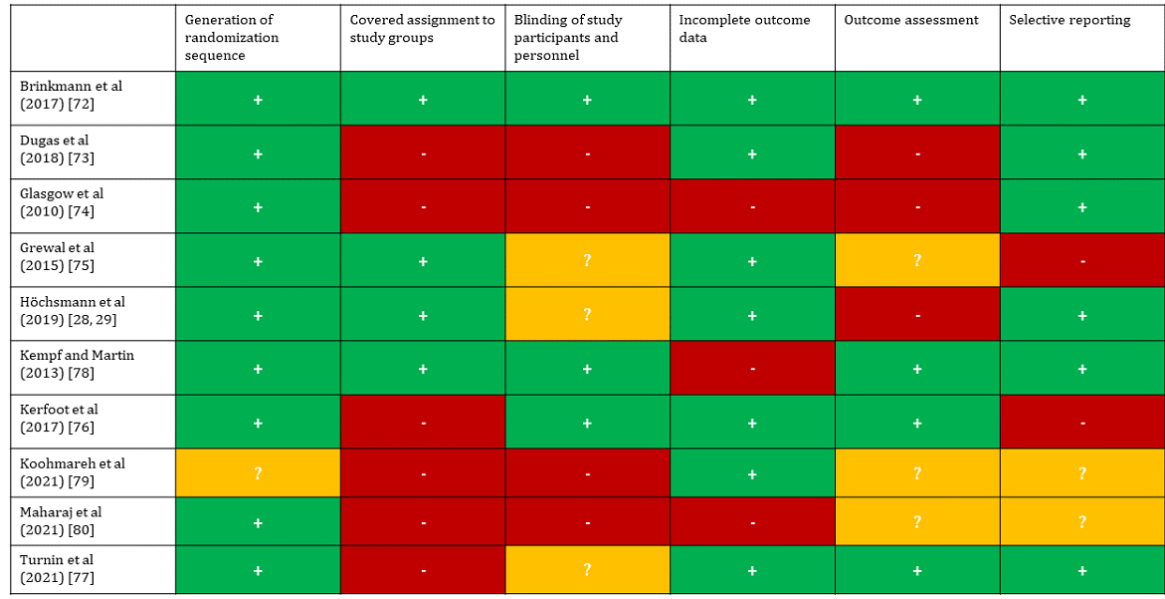
Results of the quality assessment applying the Cochrane risk-of-bias tool version 2 [28,29,72,-80].

Applying the RoB 2, a high risk of bias was detected in 90% (9/10) of the included RCTs [28,73-80]. A total of 10% (1/10) of the studies had a low risk of bias [72]. For 40% (4/10) of the RCTs, multiple reasons for a high risk of bias were detected [73,74,79,80], whereas for 30% (3/10) of the studies, only 1 reason was found [75,77,78]. The allocation sequence was random in all but 10% (1/10) of the cases [79]. Major sources of bias were the blinding of study participants and personnel to the allocation to either the intervention or the control group [73,74,76,77,79,80] and, to a smaller degree, inadequate (ie, nonreliable or nonvalidated) measures for outcome assessment [28,73-75,79,80], both of which translate to detection bias. Most of the included RCTs (7/10, 70%) conducted per-protocol analyses [28,29,72,73,75,77,78,80], whereas another study did not specify the type of analysis [79]. As such, the effect estimate was potentially biased by dropouts in all but 20% (2/10) [74,76] of the included RCTs (attrition bias). Glasgow et al [74] and Kerfoot et al [76] performed an intention-to-treat analysis, whereas the remaining authors all performed per-protocol analyses except for Koohmareh et al [79], for whom the type of analysis could not be discerned. Bias because of selective reporting was detected by comparing the outcomes described in the Methods sections and those reported in the Results sections [75,76]. The results of the quality assessment can be found in Figure 2.

### Game Types and Components Analyzed in the Included RCTs

Of the 10 included RCTs, 4 (40%) focused on an exergame [28,29,72,75,78], one (25%) of which reported first behavioral and then clinical results of the same exergame intervention studied with the same population [28,29]. A total of 20% (2/10) of the studies reported on the effects of game components within digital self-management applications for diabetes [73,80]. In 40% (4/10) of the RCTs, the authors analyzed quiz games with DSME content [74,76,77,79]. The digital intervention studied by Brinkmann et al [72] combined an exergame with a game for cognitive problem-solving.

Within the exergames, virtual reality components such as virtual race tracks were used [72,75,78]. Scoring systems awarding trophies to winners or when individualized scores were achieved were used in 60% (6/10) of the interventions [28,29,73,74,76,79,80]. Storytelling features were part of 20% (2/10) of the interventions studied [28,79], and 10% (1/10) of the interventions applied a team-based approach to the game [78].

Among the 4 RCTs in which the authors reported on the matter [72,75,78,79], intervention intensity varied between 30 minutes once (equal to intervention duration) [72], 15 minutes per day for 6 weeks [79], 30 minutes per day for 12 weeks [78] and 45 minutes twice a week for 4 weeks [75].

A complete overview of the intervention types and game components studied in the included RCTs can be found in Table 2.

**Table 2:** Intervention characteristics of the included randomized controlled trials.

Study, year	Intervention group	Control group	Intervention	
			Duration	Intensity

Brinkmann et al [72], 2017	Exergaming: interactive video games using Wii Fit Plus combining physical activity and cognitive challenges (n=8) aiming to improve neurotrophic factors	Cycling on stationary bicycle (n=8)	30 minutes	30 minutes
Dugas et al [73], 2018	Using self-management app with game elements (point reward system for achieving target values in clinical and behavioral outcomes) aiming to improve diabetes outcomes in 4 randomized conditions: App use only (n=5) App use including provider communication features (n=5) App use including team engagement features (n=6) App use including team engagement and provider communication features (n=6)	Usual care (n=5)	13 weeks	Not reported
Glasgow et al [74], 2010	Self-management website allowing for goal setting in medication adherence, nutrition, and exercise, including quiz component (n=169) Augmented with (1) follow-up calls with a member of the study staff to ask questions about the study and receive help in handling the website and (2) group meeting with other study participants (n=162)	Enhanced usual care (automated feedback on health risks based on patient-provided disease-relevant parameters; recommendation of preventive behaviors; n=132)	16 weeks	Not reported
Grewal et al [75], 2015	Virtual obstacle-crossing task with audio and video feedback on a virtual reality interface Ankle-reaching task with virtual representation and feedback onscreen (n=16)	Usual care (n=18)	4 weeks	45 minutes twice a week
Höchsmann et al [28,29], 2019	Smartphone game “Mission: Schweinehund” based on self-determination theory and including behavioral or motivational elements as well as a storyline where users restore a garden with resources gained through individualized in-game workouts tracked via phone sensors (n=18)	1-time lifestyle counseling and structured exercise plan (n=18)	24 weeks	Not reported
Kempf and Martin [78], 2013	Wii Fit Plus including balance board (n=120)	Routine care (n=100)	12 weeks	>30 minutes per day for 12 weeks
Kerfoot et al [76], 2017	Team-based mobile DSME^a^ quiz with scoring system plus booklet on civic issues (n=227)	Team-based mobile quiz on civic issues plus DSME booklet (n=229)	24 weeks	Not reported
Koohmareh et al [79], 2020	Amoo mobile game—glycemic index and calorie training game (n=30)	Educational content similar to the game through a brochure (n=30)	6 weeks	15 minutes per day for 6 weeks
Maharaj et al [80], 2021	mySugr—self-monitoring and self-management app with nudging and game component (point reward system for certain behaviors; n=44)	Glucose Buddy—self-monitoring and self-management app (n=45)	2 weeks	Not reported
Turnin et al [77], 2021	DSME software including quiz components (n=135)	Usual care (n=128)	48 weeks	Not reported

^a^DSME: Diabetes Self-Management and Education.

### Effects of Gamified Health Interventions on Health Behavior

Exercise intensity and adherence were studied in 50% (5/10) of the included studies [28,29,73-75,78]. A total of 40% (4/10) of the studies reported on nutritional behavior [73,74,79,80], and 20% (2/10) of the studies reported on medication adherence [73,74].

Positive effects on behavioral PA outcomes were reported in several of the included RCTs (4/10, 40%). A total of 50% (2/4) of the exergames studied led to an increased intensity of PA (measured as step count, caloric expenditure, and self-report) in the study participants [28,29,78], and so did the quiz component nested within the DSME website studied by Glasgow et al [74]. However, the latter was only effective when the website was supplemented with follow-up calls by study staff as a supportive measure in handling the website and group meetings with other study participants [74]. Only Höchsmann et al [28] reported on PA adherence and found no substantial effect of their exergame on PA adherence, where PA exercises were nested within a coherent narrative. However, the intervention statistically significantly increased intrinsic motivation for PA (*P*<.001), whereas intrinsic motivation decreased in the control group (1-time lifestyle counseling and structured exercise plan) during the study period. Between-group effects were significant. The authors also found a significant positive relationship between time spent doing the in-game exercises and PA motivation (*P*=.01) [28].

Dugas et al [73] found no overall intervention effect of any of the intervention arms studied on adherence as a whole (quantified using an in-app rating system; Table 3), but they found a statistically significant interaction effect (in regression analysis) of time spent using the self-management app with game elements and assessment (*P*=.01). They also found a statistically significant interaction effect of time and locomotion on weekly exercise adherence (*P*<.05) [73].

In 40% (4/10) of the RCTs, the authors found significant positive effects on *nutrition behavior*. Glasgow et al [74] reported a significant decrease in fat intake in the intervention group compared with that in the control group (*P* value for intergroup differences=.006). Koohmareh et al [79] found that participants using their educational game on glycemic index and calorie intake paid significantly more attention to the glycemic levels and calorie count of their food (*P* value for intergroup differences=.001). Maharaj et al [80] showed that participants using the mySugr app with the reward system could significantly reduce their high-fat food consumption, whereas no significant changes were found in the control group using a self-management app without a game component (*P* value for intergroup differences=.052). Interaction effects of time and assessment as well as of time and locomotion were found by Dugas et al [73] for nutrition adherence (quantified using an in-app rating system) as well. The authors also found a significant positive effect of locomotion on nutritional adherence (*P*<.05) but none of assessment (*P* value not reported) [73].

Glasgow et al [74] found no significant effect of the intervention on medication adherence or on eating habits other than fat intake (*P* value for intergroup differences=.006). Dugas et al [73] found an interaction effect of time spent using the intervention and assessment on medication adherence (*P*=.04).

A total of 40% (2/5) of the exergames studied had no effect on PA intensity [75,78]. Overall, positive effects of digital health interventions with game components could be found on motivation for and intensity of PA as well as on eating habits.

**Table 3:** Results of the included randomized controlled trials according to all relevant outcomes and overall study quality.

Study, year	Outcome (outcome measure)	Results			Study quality according to RoB 2^a^
		Statistically significant	Nonsignificant		
Brinkmann et al [72], 2017	BDNF^b^, VEGF^c^, and IGF-1^d^ (all measured using an enzyme-linked immunosorbent assay) Heart rate Lactate values	Clinical parameters: Significantly better lactate values in intervention group (*P*=.04)	Clinical parameters: Insignificant lower heart rate in intervention group (*P*>.05) Insignificant increase in BDNF in intervention group (*P* value not reported) Insignificant increase in VEGF in intervention group (*P* value not reported) Insignificant increase in IGF-1 in intervention group (*P* value not reported)	High	
Dugas et al [73], 2018	HbA_1c_^e^ Regulatory mode (locomotion and assessment scales developed by Kruglanski et al [81]) Adherence to healthy behaviors (glucose, medication, and nutrition tracking entered manually into app; PA^f^ tracked via Fitbit or manually; and recorded app use, all quantified using in-app point rating system)	Behavioral outcomes: Significant positive causal relationship between locomotion and weekly adherence score (*P*<.05) Significant positive causal effect of interaction between time and assessment during intervention time on adherence (*P*=.01) Significant positive causal effect of interaction between time and locomotion for app use, including provider communication features, on adherence (*P*=.04) Clinical parameters: Significant positive causal effect of app use, including provider communication features, on HbA_1c_ (*P*<.01) Significant positive causal effect of interaction between time and adherence on HbA_1c_ (*P*<.01)	Behavioral outcomes: No treatment effect of any intervention arm on total adherence (*P* value not reported) Clinical parameters: No significant between-group differences in HbA_1c_ during intervention time (*P* value not reported)	Low	
Glasgow et al [74], 2010	Health literacy (brief questionnaire by Chew et al) Dietary behavior (“Starting the Conversation Scale” by Ammerman et al) Adherence to medication for diabetes, blood pressure, and cholesterol (medication-taking items of the Hill-Bone Compliance Scale) Fat intake (National Cancer Institute Percent Energy from Fat Screener) Total weekly caloric expenditure (Community Healthy Activities Model Program for Seniors Questionnaire) HbA_1c_ BMI^g^ Lipid ratio Mean arterial pressure	Behavioral outcomes: Significant improvement in eating habits in intervention group but not in control group (*P* value for intergroup differences ≤.001) Significant decrease in fat intake in intervention group but not in control group (*P* value for intergroup differences=.006) Significant increase in PA in intervention group but not in control group (*P* value for intergroup differences=.04)	Behavioral outcomes: No significant improvement in medication adherence in intervention group vs control group (*P* value for intergroup differences=.29) No significant differences in eating habits (*P* value for intergroup differences=.08), fat intake (*P* value for intergroup differences *P*=.46), PA (*P* value for intergroup differences=.63), and medication adherence (*P* value for intergroup differences=.86) between intervention groups PROs^h^: No interaction effect of health literacy on any of the outcomes measured (P not reported) Clinical parameters: No significant improvement of HbA_1c_ (*P* value for intergroup differences=.42), BMI (*P* value for intergroup differences=.19), lipid ratio (*P* value for intergroup differences=.90), and mean arterial pressure (*P* value for intergroup differences=.83) in any study arm	Low	
Grewal et al [75], 2015	Postural stability (FES-I^i^ and postural stability) Diabetes peripheral neuropathy (VPT^j^) Daily PA (time spent sitting, standing, and walking and total step count) measured via shirt-worn sensor Quality of life (SF-12^k^)	PROs: Significant improvement in mental health component of SF-12 in intervention group but not in control group (*P* value for intergroup differences=.04) Clinical outcomes: Significant reduction in average center of mass sway area (in degrees) in intervention group but not in the control group (*P* value for intergroup differences=.009) Significant reduction in medial-lateral center of mass sway area in intervention group but not in the control group (*P* value for intergroup differences=.008) Significant reduction of hip and ankle sway in the intervention group but not in the control group (*P* value for intergroup differences=.008) Significant reduction of ankle sway degree in intervention group but not in control group (with and without blindfold; *P* value for intergroup differences=.02)	Behavioral outcomes: No significant differences in time spent sitting (*P*=.62), standing (*P*=.36), or walking (*P*=.08) in intervention and control group PROs: No significant differences in physical component of SF-12 in intervention and control group (*P* value for intergroup differences=.64) No significant differences in FES-I in intervention and control group (*P* value for intergroup differences=.31) Clinical outcomes: No significant reduction in anterior-posterior center of mass sway area in intervention or control group (*P* value for intergroup differences=.38) No significant reduction in average center of mass sway area in intervention or control group when blindfolded (*P* value for intergroup differences=.18) No effects on VPT reported	Low	
Höchsmann et al [28,29], 2019	Intrinsic PA motivation (12-item version of the Intrinsic Motivation Inventory) PA adherence (step count, stride cadence, completed vs canceled in-game workouts, and duration and patterns of game use) HbA_1c_ Aerobic capacity (cardiorespiratory fitness [maximum oxygen uptake and first ventilatory threshold]) Daily PA (step count via accelerometer wristband) Total cholesterol LDL-C^l^ HDL-C^m^ Triglycerides Resting heart rate SBP^n^ DBP^o^	Behavioral outcomes: Significant increase in intrinsic PA motivation in the intervention group (*P*<.001) and nonsignificant decline in the control group (*P*>.05) Significant between-group differences in intrinsic PA motivation after intervention (*P*<.05) Significant between-group differences in intrinsic PA motivation after intervention on the subscales for interest or enjoyment (*P*<.05) and perceived competence (*P*<.05) (significant increase in intervention group (*P*<.001) Significant increase in intrinsic PA motivation after intervention on the subscale for perceived choice in the intervention group (*P*<.05) but no between-group differences (*P*>.05) Significant relationship between in-game exercise (measured in minutes) and changes in intrinsic PA motivation (total score) (*P*=.01) Daily PA increase in the intervention and control group with a significant difference between groups (*P*<.001) Only descriptive reporting of PA adherence Clinical parameters: Significantly higher increase in step count in intervention group than in control group (*P*<.001) No changes in HbA_1c_ in the intervention group but increase in the control group (significant between-group differences) (*P*=.02) Significant increase in aerobic capacity in the intervention group and decrease in the control group (significant between-group differences) (*P*<.001) Significantly higher decrease in body fat mass in intervention than in control group (*P*=.045)	Behavioral outcomes: Nonsignificant increase in intrinsic PA motivation after intervention on the subscale for value/usefulness (*P*>.05) but significant between-group differences (*P*<.05) Only descriptive reporting of PA adherence Clinical parameters: No changes in resting heart rate (*P*=10), SBP (*P*=.38), and DBP (*P*=.18) in any of the study groups No changes in skeletal muscle mass in any of the study groups (*P*=.71) No changes in total cholesterol (*P*=.55), HDL-C (*P*=.46), LDL-C (*P*=.74), and triglycerides (*P*=.95) in any of the study groups	Low [28] Moderate [29]	
Kempf and Martin [78], 2013	HbA_1c_ BMI FPG^p^ Total cholesterol LDL-C HDL-C Triglycerides Self-reported PA SBP DBP Self-assessed diabetes-related impairment using PAID^q^ Self-assessed physical and mental well-being using SF-12 Subjective well-being using WHO-5^r^ Quality of life using ADS-L^s^	Behavioral outcomes: Significant increase in PA in both groups (*P* value for intergroup differences <.001) PROs: Significant decrease in diabetes-related impairment in both groups (*P* value for intergroup differences=.03) Significant improvement in subjective well-being in the intervention group but not in the control group (*P* value for intergroup differences=.004) Significant improvement in mental health in the intervention group but not in the control group (significant between-group effects; *P* value for intergroup differences=.02) Significant improvement in quality of life in the intervention group but not in the control group (significant between-group effects; *P* value for intergroup differences <.001) Clinical parameters: Significant reduction in HbA_1c_ in the intervention group, but not in the control group (*P* value for intergroup differences<.001) Significant reduction in BMI in both groups (*P* value for intergroup differences=.008) Significant reduction in weight in both groups (*P* value for intergroup differences <.001) Significant decrease in FPG in both groups (*P* value for intergroup differences=.008)	Clinical parameters: No significant differences in DBP, SBP, total cholesterol, HDL-C, LDL-C, triglycerides, metformin or DPP-4^t^ inhibitor treatment, or physical well-being in any of the study groups (*P* value not reported)	Low	
Kerfoot et al [76], 2017	HbA_1c_ Oral diabetes medication PPR^u^ Urine microalbumin to creatinine ratio Diabetes Empowerment Scale-Short Form Self-assessed diabetes-related impairment using PAID	PROs: Significant increase in empowerment in the intervention group but decrease in the control group during intervention time (significant between-group differences; *P* value for intergroup differences=.01) Clinical parameters: Significantly higher reduction in HbA_1c_ in the intervention group than in the control group both after the intervention and at follow-up (significant between-group differences; *P* value for intergroup differences=.048) Significantly higher reduction in HbA_1c_ in the intervention group for patients with baseline HbA_1c_ of >75 mmol/mol (*P*=.03)	Behavioral outcomes: No significant differences in PPR in any group (*P* value not reported) PROs: No significant differences in diabetes-related impairment during intervention time in any study group (*P* value not reported) No significant differences in diabetes-related impairment and empowerment at follow-up in any study group (*P* value not reported) Clinical parameters: No significant differences in urine microalbumin to creatinine ratio in any study group (*P* value not reported)	Low	
Koohmareh et al [79], 2020	FPG Knowledge of diabetes diet (self-developed Amoo^v^ test) Attention to food glucose levels and food calories	Behavioral outcomes: Significantly more attention to food glucose levels and food calories in intervention group but not in the control group (*P* value for intergroup differences=.001) PROs: Significantly higher Amoo test scores in the intervention group but not in the control group (*P* value for intergroup differences=.001)	Clinical outcomes: No significant differences in FPG (*P* value for intergroup differences=.63)	Low	
Maharaj et al [80], 2021	Self-care behaviors (SDSCA^w^) Illness beliefs (Brief Illness Perception Questionnaire)	Behavioral outcomes: Borderline significant median difference in high-fat food consumption (lower in mySugr group; *P* value for intergroup differences=.052)	Behavioral outcomes: No significant median differences in self-care behaviors in any study group (*P* value for intergroup differences=.64) PROs: No significant median differences in illness beliefs in any study group (*P* value for intergroup differences=.05)	Low	
Turnin et al [77], 2021	HbA_1c_ BMI Waist circumference	Clinical outcomes: Significant reduction in waist circumference in intervention group but not in control group (*P* value for intergroup differences=.04) Stronger intergroup differences in frequent users (*P*=.008)	Clinical outcomes: No significant differences in HbA_1c_ between intervention and control group after adjustment for risk factors (*P* value for intergroup differences=.12) No significant differences in BMI between intervention and control group after adjustment for risk factors (*P* value for intergroup differences=.08)	Low	

^a^RoB 2: Cochrane risk-of-bias tool version 2.

^b^BDNF: brain-derived neurotrophic factor.

^c^VEGF: vascular endothelial growth factor.

^d^IGF-1: insulin-like growth factor–1.

^e^HbA_1c_: glycated hemoglobin (blood sugar value).

^f^PA: physical activity.

^g^BMI: body mass index

^h^PRO: patient-reported outcome.

^i^FES-I: Falls Efficacy Scale–International.

^j^VPT: vibration perception threshold.

^k^SF-12: Short Form Health Survey.

^l^LDL-C: low-density lipoprotein cholesterol.

^m^HDL-C: high-density lipoprotein cholesterol.

^n^SBP: systolic blood pressure.

^0^DBP: diastolic blood pressure.

^p^FPG: fasting plasma glucose.

^q^PAID: Problem Areas in Diabetes Scale.

^r^WHO-5: 5-item World Health Organization Well-Being Index.

^s^ADS-L: *Allgemeine Depressionsskala* long version (German).

^t^DPP-4: dipeptidyl peptidase 4.

^u^PPR: patient-pill ratio.

^v^Amoo: diabetes test designed by Koohmareh et al [79].

^w^SDSCA: Summary of Diabetes Self-Care Activities.

### Effects of Gamified Health Interventions on PROs

In the included RCTs, data were provided on health literacy in general [74,79,80], quality of life [75,78], diabetes-related impairment [76,78], and subjective well-being [78].

Concerning *health literacy*, Koohmareh et al [79] found a significant positive effect of their educational game on knowledge concerning a diet adequate for patients with diabetes (measured on a self-developed scale; *P*=.001). Maharaj et al [80] found no significant median differences in illness beliefs (measured using the Brief Illness Perception Questionnaire) between the gamified mySugr and the self-management app without any game components (*P* value for intergroup differences=.05). Glasgow et al [74] used health literacy as a moderating variable in a multivariate analysis of covariance to measure the effects of their self-management website with quiz elements but found no interaction effects (*P* value not reported).

In the domain of *quality of life*, Grewal et al [75] found a significant improvement in mental well-being because of the virtual balance training they studied (*P* value for intergroup differences=.04) but no effect of said intervention on physical well-being (*P* value for intergroup differences=.64; both measured using the Short Form 12 Health Survey). The same effect was demonstrated by Kempf and Martin [78], who studied Wii Fit Plus games, using both the Short Form 12 Health Survey and a German depression scale. *Subjective well-being*, measured using the 5-item World Health Organization Well-Being Index scale, improved significantly in the study by Kempf and Martin [78] as well (*P* value for intergroup differences=.004).

Grewal et al [75] and Kempf and Martin [78] studied the effects of gamified interventions on *diabetes-related impairment* both using the Problem Areas in Diabetes Scale. Although the latter found a positive effect of the Wii Fit Plus games (*P* value for intergroup differences=.03) [78], the former found no effect of virtual balance training (*P* value not reported) [75]. In terms of impairment, Grewal et al [75] found no effect of the virtual balance training on fear of falling (*P* value for intergroup differences=.31).

Overall, sparse positive effects of digital health interventions with game components could be found on health literacy and diabetes-related impairment, whereas substantial evidence was found for the improvement of subjective mental well-being.

### Effects of Gamified Health Interventions on Clinical Outcomes

The clinical outcome studied most often in the included RCTs was *HbA_1c_* [29,73,74,76-78]. Furthermore, 30% (3/10) of the included studies analyzed changes in BMI [74,77,78], whereas Höchsmann et al [28], Glasgow et al [74], and Kempf and Martin [78] also analyzed lipid outcomes and blood pressure values. Fasting plasma glucose (FPG) was studied twice [78,79], and so was the heart rate of the participants [28,72]. Brinkmann et al [72] studied cognitive parameters in conjunction with lactate values, and Grewal et al [75] analyzed postural stability.

**Figure 3:**
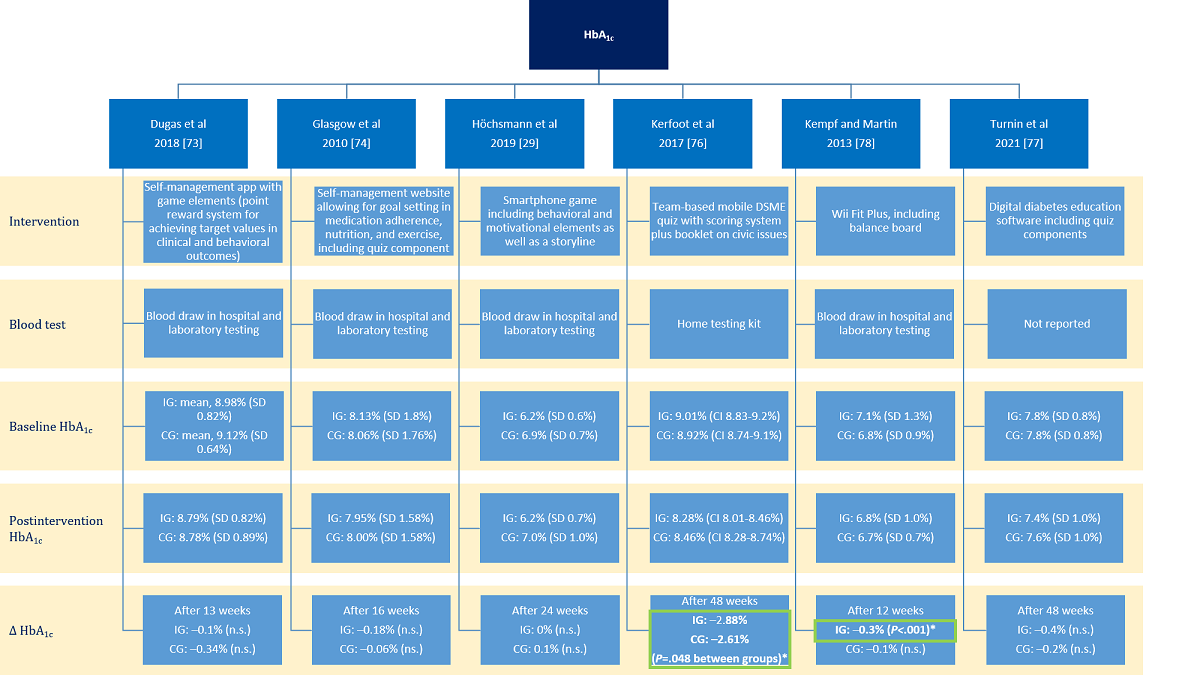
Effects of the interventions studied on glycated hemoglobin (HbA_1c_) levels. *Significant. Δ HbA_1c_: difference in HbA_1c_ between pre- and postintervention measurement; CG: control group; DSME: Diabetes Self-Management and Education; IG: intervention group; n.s.: not significant [29,73,74,76-78].

The effects of the gamified interventions on HbA_1c_ levels studied in 60% (6/10) of the RCTs are depicted in Figure 3, where asterisks mark significant changes in HbA_1c_. Only Kerfoot et al [76] (*P* value for intergroup differences=.048) and Kempf and Martin [78] (*P* value for intergroup differences<.001) found significant positive effects of a mobile, team-based DSME quiz and Wii Fit Plus games, respectively, on HbA_1c_ levels in terms of a reduction in the intervention group and significant between-group effects. Dugas et al [73] found a significant effect of time spent using the self-management app with an award system on HbA_1c_ levels in a regression analysis (*P*<.01), as well as an interaction effect of total adherence score (additive score of exercise, nutrition, and medication adherence; *P*<.01).

Kempf and Martin [78] also found significant positive effects of the intervention on *BMI* (*P* value for intergroup differences=.008); however, Glasgow et al [74] (*P* value for intergroup differences=.20) and Turnin et al [77] (*P* value for intergroup differences=.08), also studying DSME software with quiz components, did not. Significant effects on *FPG* after using the same software were also reported by Kempf and Martin [78] (*P* value for intergroup differences=.008) but not by Koohmareh et al [79] (*P* value for intergroup differences=.63), who studied the same intervention type. Significant effects were not found on lipid outcomes and blood pressure values [29,74,78] or on cognitive outcomes and lactate values [72]. Grewal et al [75] found their exergame, including a virtual obstacle course, to have a positive effect on postural stability when compared with usual care (*P* value for intergroup differences=.009).

Turnin et al [77] found a significant reduction in *waist circumference* in the intervention group when compared with the control group both before and after adjusting for confounding factors such as baseline HbA_1c_ levels, age, sex, or obesity (*P* value for intergroup differences=.04). The authors also reported an increased effect on waist circumference in those with a higher frequency of use (*P*=.008).

No significant effects were found on *heart rate* [29,72], *blood pressure* (systolic or diastolic) [29,78], or *lipid values* (ie, on total cholesterol, triglycerides, low-density lipoprotein cholesterol, or high-density lipoprotein cholesterol) [29,78] in any of the RCTs that reported on these values.

Brinkmann et al [72] studied the vascular endothelial growth factor as an indicator of *diabetes retinopathy* but found no effect of the Wii Fit exergame they studied (*P* value not reported). Furthermore, they found no effect of the intervention on insulin-like growth factor–1 as a proxy for *poorly adjusted diabetes* (*P* value not reported).

Brinkmann et al [72] found improved lactate values in the intervention group (*P*=.04) but not in the control group (*P* value not reported), which used a stationary bicycle, thus indicating higher *fitness* levels owing to the exergame. Improved fitness because of an exergame was also demonstrated by Höchsmann et al [29] using aerobic capacity as an indicator (*P*<.001). Grewal et al [75] found improved postural stability to be an effect of the virtual balance training they studied (*P*=.009).

As Brinkmann et al [72] aimed to also improve *cognitive functioning* of the study participants by adding cognitive challenges to the exergame, they studied the brain-derived neurotrophic factor as an indicator of learning and memory capacity and found no significant effect of the intervention (*P* value not reported).

Overall, sparse positive effects of digital health interventions with game components could be found on clinical outcomes, with fitness-related outcomes improving the most.

A complete overview of the effects found in the included RCTs can be found in Table 3. If not stated otherwise, differences in outcomes are reported between the intervention and control group(s) before and after the study in Table 3.

## Discussion

### Principal Findings

Despite heterogeneity in the effects of the gamified applications studied in this review on behavioral, clinical, and patient-reported outcomes, certain patterns emerged. Exergames had the tendency to improve fitness-related and, to a smaller extent, clinical values (as shown in 4/10, 40% of the RCTs [73,76-78]), whereas educational games affected disease-related knowledge and especially nutrition behavior (as shown in 4/10, 40% of the RCTs [73,74,79,80]). In addition, exergames had a potential to improve outcomes related to self-reported well-being such as quality of life and diabetes-related impairment (as shown in 2/10, 20% of the RCTs [75,78]).

### Comparison With Prior Work

The results confirm those of a meta-analysis by DeSmet et al [82], who showed significant positive effects of serious games on health behavior independent of any diagnosis and its theory-based determinants and significant but much smaller effects on various clinical outcomes. As for nutrition behavior, which improved according to 40% (4/10) of the RCTs included in this review solely because of self-management applications with quiz elements, Ledoux et al [83] found similar effects of a serious game for young patients with type 1 diabetes.

Somewhat surprisingly, self-efficacy was not among the outcomes studied in the included RCTs despite existing evidence that both exergames and serious games can increase the feeling of being able to actively affect one’s health outcomes [65,84]. A precondition derived from behavior change theories is the option for goal setting by the intervention participants themselves as opposed to behavioral goals predefined by the intervention developers [85]. This precondition was met by all the gamified interventions studied in this review that used a reward or scoring system as these game components allow for the autonomous setting of target values that the users aim to achieve and, thus, generate intrinsic motivation [12].

A coherent narrative as an instrument of storytelling was used in only 20% (2/10) of the included interventions; however, it aided in producing positive effects on behavioral, knowledge-related [28,79], and clinical outcomes [29]. As such, the results confirm observations made for narrative health communication messages when delivered in a digital manner [86].

Physical fitness because of an increase in PA owing to interventions with game components is an especially promising result as PA is one of the primary target behaviors of diabetes self-management. The same is true for dietary behavior [6]. As such, these results underline once more the potential of digital interventions for DSME, acknowledged also by the ADA and the European Association for the Study of Diabetes in a joint statement [87].

The relatively high mean age of the study participants is surprising insofar as the traditional target group for digital health applications of all sorts is usually younger people [88]. Christensen et al [17], in sensitivity analyses, also found a positive effect of game-based interventions only for people aged <18 years. Especially regarding exergaming, the positive effects for older people because of high engagement have already been proven elsewhere [89]. Given the fact that most of the gamified interventions studied in this review (7/10, 70%) were designed for mobile devices, the high penetration of such devices in all age groups might play an important role in overcoming the digital health divide because of age [90]. Therefore, the results need to be considered in light of demographic changes and a rise in the demand for health care, especially for chronic diseases [91].

With intervention duration varying widely (Table 2), statements on its relevance have to be made with caution. However, the results show that effects on clinical outcomes can be achieved via digital health interventions with game components after at least 12 months [76,78], which corresponds to the concept of DSME as a continuous, long-term intervention [4,6]. However, even 36 weeks of intervention duration did not guarantee significant effects on BMI and HbA_1c_ [74], hinting at a washout in intervention fidelity common in digital health applications [92].

The fact that generally positive effects of digital health interventions of any kind are biased because of low overall study quality also mirrors findings of recent evidence syntheses [22,93]. The methodological issues concerning the quality of obtainable evidence raised in the joint statement by the ADA and the European Association for the Study of Diabetes [87] persist in this review even though all the included studies (10/10, 100%) were RCTs or had adaptive RCT designs. The issue of blinding of study participants and personnel to the allocation is common in digital health trials as the fact that one did not receive a gamified health application is easily uncovered [8]. Therefore, the study conducted by Maharaj et al [80] is a positive example of comparing 2 digital health applications whereby one is augmented with game components. The issue of missing outcome data because of dropouts, well known to researchers in multiple fields, is also common in digital health research [94] and is linked to the issue of intervention fidelity. The reasons as to why trial participants may lose interest in the use of digital health applications can be numerous [92]. Therefore, participatory design is an important precondition for gamified applications as well [95]. Nonvalidated outcome measures, another major source of bias in the included RCTs, point to the need to develop core outcome sets for digital health applications with or without game components.

### Strengths and Limitations

Robust and reproducible systematic review methods were used to identify the best available evidence, and the results were reported according to the PRISMA checklist (Multimedia Appendix 3 [23]). Owing to the fact that all steps taken in this review were performed by 2 researchers independently and checked by a third researcher, it is highly unlikely that relevant records were overlooked or incorrectly discarded as irrelevant. The same is true for information in the included records relevant to the quality assessment.

The focus on research published in German or English is a limitation. Owing to the diverse intervention types (with intervention durations varying widely) and outcomes studied in the rather large number of included RCTs (compared, eg, with the meta-analysis by Christensen et al [17]), a meta-analysis especially of clinical outcomes was not deemed feasible. Rather, the broad realm of outcomes studied allows for a holistic overview of the potential effects of digital health interventions with game components.

Some of the included RCTs (3/10, 30%) had a considerable loss to follow-up, which would have required intention-to-treat analyses. However, most studies (7/10, 70%) conducted per-protocol analyses instead. Furthermore, the reasons for dropout were not always reported.

Little to no follow-up time after the intervention period limits the comparability of the gamified interventions studied in the included RCTs with other digital or analog behavior change interventions that have demonstrated sustainable effects over time [96]. However, according to both theory and evidence, sustainable and long-lasting behavior change over a time span of at least 6 months is necessary to achieve improved health outcomes and still not easily achieved [85]. Owing to limited reporting of intervention intensity, no discernible patterns regarding effectiveness could be found. Moreover, effective components of gamified health interventions regarding both clinical outcomes and behavior change have not been identified yet [97].

### Conclusions

This systematic review provided a thorough analysis of the effectiveness of digital health interventions with game components for the self-management of type 2 diabetes. The included RCTs analyzing exergames showed positive effects on fitness-related outcomes and, albeit only in 1 case, also on HbA_1c_. Educational games improved dietary habits and subjective mental health and well-being. However, the evidence base was ambiguous and further limited because of the considerable risk of bias in the study designs of most of the included RCTs (9/10, 90%). Nevertheless, the results imply that digital health interventions with game components can help improve PA, dietary habits, and well-being. Therefore, these applications, when developed based on theory and evaluated rigorously, can help achieve the behavioral goals mentioned in several guidelines for DSME. Given the mostly low quality of the included RCTs, the presented evidence allows for nothing more than the suggestion of digital health interventions with game components as a supplement to traditional DSME, which corresponds to the statement by Fleming et al [87]. The main results of this review are summarized in Textbox 3.

**Main results of the review.**

- The variety of game components used in digital health interventions for type 2 diabetes ranges from quiz components over storytelling elements to full-scale exergames.
- Digital health interventions containing game components might improve the health behavior patterns, quality of life, and clinical outcomes of patients with type 2 diabetes.
- The methodological quality was low in most of the included randomized controlled trials.

Further research should compare digital health interventions that contain game components with those that do not rather than comparing the former with usual care and, as such, risking bias because of high dropout rates. Furthermore, longer follow-up assessments are crucial to detect whether the effects of gamified interventions are sustainable. Adaptive study designs such as microrandomization, where individuals are randomized to several intervention components or treatments for the study duration [98], could be used to determine which game components have an effect on which outcome domain (behavioral, PROs, or clinical). Larger sample sizes would allow for more detailed subgroup analyses [99] to determine which user groups, in addition to those defined by age, profit the most from digital health interventions containing game components. Finally, but importantly, a comparison of the effects of digital health interventions with game components for patients with type 1 and 2 diabetes is bound to provide further insights.

## Appendix

Multimedia Appendix 1 Database-specific search strings for PubMed and PsycINFO.

Multimedia Appendix 2 List of excluded studies with reasons.

Multimedia Appendix 3 PRISMA (Preferred Reporting Items for Systematic Reviews and Meta-Analyses) checklist.

## Abbreviations

ADA: American Diabetes Association
CASP: Critical Appraisal Skills Programme
DSME: Diabetes Self-Management and Education
FPG: fasting plasma glucose
HbA_1c_: glycated hemoglobin
PA: physical activity
PRISMA: Preferred Reporting Items for Systematic Reviews and Meta-Analyses
PRO: patient-reported outcome
RCT: randomized controlled trial
RoB 2: Cochrane risk-of-bias tool version 2
