# Supplementary material for: Effectiveness of Digital Health Interventions Containing Game Components for the Self-management of Type 2 Diabetes: Systematic Review
Source: JMIR Serious Games. 2023 Jun 1;11:e44132. doi: 10.2196/44132 (PMC10273035; doi:10.2196/44132)
Supplement: Multimedia Appendix 1 [file games_v11i1e44132_app1.docx]

**Appendix 1 – Database-specific search strings**

*Search String for PubMed*

| Population | (("Diabetes mellitus type two" OR "diabetes type two" OR "Blood Glucose"  [Mesh] OR "Blood Glucose Self-Monitoring" [Mesh] OR "Hyperglycemia" [Mesh] OR Hyperglycem* OR "Hypoglycemia" [Mesh] OR Hypoglycem* OR "Glycated Hemoglobin A" [Mesh] OR "Glucose Intolerance" [Mesh] OR IGT OR "Impaired Glucose Tolerance" OR IFG OR "Impaired Fasting Glucose" OR "blood glucose" OR diabetes OR "Impaired blood glucose" OR "Diabetes mellitus type 2" OR "Diabetes mellitus type II" OR "type II Diabetes" OR "type two Diabetes" OR "Type 2 Diabetes" OR "Diabetes type 2" OR "Diabetes type II" OR "Diabetes Mellitus, Type 2" [Mesh] OR T2D OR T2DM OR dm2 OR iddm OR niddm OR "non-insulin dependent" OR "noninsulin dependent" OR "insulin dependent" OR (type ii diabet*))) |
| --- | --- |
| AND |  |
| Intervention | (interactive OR "interactive game" OR "interactive games" OR "interactive gaming" OR "interactive learning" OR "simulated environment" OR "educational game" OR "educational games" OR "educational gaming" OR "digital game" OR "digital games" OR "digital gaming" OR "digital interventions" OR gamification OR game* OR gaming OR exergam* OR "virtual reality" OR "virtual realities" OR "virtual reality exposure therapy" OR "virtual world" OR "virtual worlds" OR VR OR "active video" OR "active videos" OR "game-based" OR "serious gaming" OR "serious game" OR "serious games" OR "collection system" OR "collection systems" OR badge* OR "virtual good" OR "virtual goods" OR "experience point" OR "experience points" OR wii OR Nintendo OR narrative OR "virtual story" OR "virtual stories" OR trophy OR trophies OR medal OR medals OR stamp OR stamps OR award OR awards OR leaderboard OR scoreboard OR "score board" OR "score-board" OR avatar OR avatars OR "virtual character" OR "virtual characters" OR mission OR missions OR quest OR quests OR "mobile applications" OR mHealth OR "mobile health") |
| NOT | |
| Addresses OR Autobiography OR Bibliography OR Biography OR Case Report OR Clinical Conference OR Collected Works OR Congresses OR Consensus Development Conference OR Consensus Development Conference, NIH OR Dataset OR Dictionary OR Directory OR Duplicate Publication OR Editorial OR Expression of Concern OR Festschrift OR Government Publications OR Guideline OR Historical Article OR Interactive Tutorial OR Interview OR Introductory Journal Article OR Lectures OR Legal Cases OR Legislation OR Letter OR Meta-Analysis OR News OR Newspaper Article OR Overall OR Patient Education Handout OR Periodical Index OR Personal Narratives OR Portraits OR Practice Guideline OR Publication Components OR Publication Formats OR Publication Type Category OR Research Support, American Recovery and Reinvestment Act OR Research Support, N.I.H., Extramural OR Research Support, N.I.H., Intramural OR Research Support, Non- U.S. Gov't Research Support, U.S. Gov't, Non-P.H.S. OR Research Support, U.S. Gov't, P.H.S. OR Review OR Scientific Integrity Review OR Study Characteristics OR Support of Research OR Twin Study OR Validation Studies OR Video-Audio Media OR Webcasts | |

*Search String for PsychInfo*

| Population | ( (((MM "Blood Sugar") OR (MM "Hyperglycemia")) OR (MM  "Hypoglycemia")) OR (MM "Type 2 Diabetes") ) OR ( "Diabetes mellitus type two" OR "diabetes type two" OR "Blood Glucose Self-Monitoring" OR Hyperglycem* OR Hypoglycem* OR "Glycated Hemoglobin A" OR "Glucose Intolerance" OR IGT OR "Impaired Glucose Tolerance" OR IFG OR "Impaired Fasting Glucose" OR "blood glucose" OR diabetes OR "Impaired blood glucose" OR "Diabetes mellitus type 2" OR "Diabetes mellitus type II" OR "type II Diabetes" OR "type two Diabetes" OR "Type 2 Diabetes" OR "Diabetes type 2" OR "Diabetes type II" OR T2D OR T2DM OR dm2 OR iddm OR niddm OR "non-insulin dependent" OR "noninsulin dependent" OR "insulin dependent" OR (type ii diabet* ) |
| --- | --- |
| AND |  |
| Intervention | (interactive OR "interactive game" OR "interactive games" OR "interactive gaming" OR "interactive learning" OR "simulated environment" OR "educational game" OR "educational games" OR "educational gaming" OR "digital game" OR "digital games" OR "digital gaming" OR "digital interventions" OR gamification OR game* OR gaming OR exergam* OR "virtual reality" OR "virtual realities" OR "virtual reality exposure therapy" OR "virtual world" OR "virtual worlds" OR VR OR "active video" OR "active videos" OR "game-based" OR "serious gaming" OR "serious game" OR "serious games" OR "collection system" OR "collection systems" OR badge* OR "virtual good" OR "virtual goods" OR "experience point" OR "experience points" OR wii OR Nintendo OR narrative OR "virtual story" OR "virtual stories" OR trophy OR trophies OR medal OR medals OR stamp OR stamps OR award OR awards OR leaderboard OR scoreboard OR "score board" OR "score-board" OR avatar OR avatars OR "virtual character" OR "virtual characters" OR mission OR missions OR quest OR quests OR "mobile applications" OR mHealth OR "mobile health") |
| NOT |  |
|  | qualitative study OR interview OR literature review OR systematic review  OR brain imaging OR focus group OR meta-analysis OR metasynthesis AND  Clinical Case Study, Clinical Trial, Empirical Study (-Experimental Replication, -Followup Study, -Longitudinal Study, -Prospective Study, - Retrospective Srudy), Field Study, Quantitative Study, Treatment Outcome, Twin Study |
