# Supplementary material for: Effectiveness of Digital Health Interventions Containing Game Components for the Self-management of Type 2 Diabetes: Systematic Review
Source: JMIR Serious Games. 2023 Jun 1;11:e44132. doi: 10.2196/44132 (PMC10273035; doi:10.2196/44132)
Supplement: Multimedia Appendix 2 [file games_v11i1e44132_app2.docx]

**Full texts exlcuded with reasons**

| Reference | Reason for exclusion |
| --- | --- |
| Saiyed, S., et al., *The Rapid Implementation of an Innovative Virtual Diabetes Boot Camp Program: Case Study.* JMIR Diabetes, 2022. **7**(1): p. e32369. [30] | Inadeqaute population |
| Owolabi, E.O., D.T. Goon, and A.I. Ajayi, *Impact of mobile phone text messaging intervention on adherence among patients with diabetes in a rural setting: A randomized controlled trial*. Medicine (Baltimore), 2020. 99(**12**): p. e18953. [31] | Inadeqaute population |
| Graham, S.A., et al., *Older Adults Engage With Personalized Digital Coaching Programs at Rates That Exceed Those of Younger Adults.* Front Digit Health, 2021. **3**: p. 642818. [32] | Inadeqaute population |
| Birse, C.E., et al., *Impact of a Digital Diabetes Prevention Program on Risk Factors for Chronic Disease in a Workforce Cohort.* J Occup Environ Med, 2020. **62**(12): p. 1040-1045. [33] | Inadeqaute population |
| Kumar, V.S., et al., *The DAILY (Daily Automated Intensive Log for Youth) Trial: A Wireless, Portable System to Improve Adherence and Glycemic Control in Youth with Diabetes.* Diabetes Technology & Therapeutics, 2004. **6**(4): p. 445-453. [34] | Inadeqaute population |
| Turnin, M.C., et al., *Evaluation of microcomputer nutritional teaching games in 1,876 children at school.* Diabetes Metab, 2001. **27**(4 Pt 1): p. 459-64. [35] | Inadeqaute population |
| Wang, J.J., et al., *Story Immersion May Be Effective in Promoting Diet and Physical Activity in Chinese Children.* Journal of Nutrition Education and Behavior, 2017. **49**(4): p. 321-329.e1. [36] | Inadeqaute population |
| Allsop, S., et al., *Comparison of short-term energy intake and appetite responses to active and seated video gaming, in 8–11-year-old boys.* British Journal of Nutrition, 2016. **115**(6): p. 1117-1125. [37] | Inadeqaute population |
| Brown, S.J., et al., *Educational video game for juvenile diabetes: results of a controlled trial.* Medical Informatics, 1997. **22**(1): p. 77-89. [38] | Inadeqaute population |
| Dring, K.J., et al., *Cytokine, glycemic, and insulinemic responses to an acute bout of games-based activity in adolescents.* Scandinavian Journal of Medicine & Science in Sports, 2019. **29**(4): p. 597-605. [39] | Inadeqaute population |
| Garde, A., et al., *Assessment of a Mobile Game (“MobileKids Monster Manor”) to Promote Physical Activity Among Children.* Games for Health Journal, 2015. **4**(2): p. 149-158. [40] | Inadeqaute population |
| Howorka, K., et al., *Empowering diabetes out-patients with structured education:: Short-term and long-term effects of functional insulin treatment on perceived control over diabetes.* Journal of Psychosomatic Research, 2000. **48**(1): p. 37-44. [41] | Inadeqaute population |
| Klaassen, R., et al., *Design and Evaluation of a Pervasive Coaching and Gamification Platform for Young Diabetes Patients.* Sensors, 2018. **18**(2). [42] | Inadeqaute population |
| Fundoiano-Hershcovitz, Y., et al., *Role of Digital Engagement in Diabetes Care Beyond Measurement: Retrospective Cohort Study.* JMIR Diabetes, 2021. **6**(1): p. e24030. [43] | Inadequate intervention |
| Guo, M., et al., *Effectiveness of mHealth management with an implantable glucose sensor and a mobile application among Chinese adults with type 2 diabetes.* J Telemed Telecare, 2021: p. 1357633x211020261. [46] | Inadequate intervention |
| Gupta, U., et al., *Effectiveness of a Video-Based Lifestyle Education Program Compared to Usual Care in Improving HbA1c and Other Metabolic Parameters in Individuals with Type 2 Diabetes: An Open-Label Parallel Arm Randomized Control Trial (RCT).* Diabetes Ther, 2020. **11**(3): p. 667-679. [47] | Inadequate intervention |
| Liu, H., et al., *Enhanced alleviation of insulin resistance via the IRS-1/Akt/FOXO1 pathway by combining quercetin and EGCG and involving miR-27a-3p and miR-96-5p.* Free Radic Biol Med, 2022. **181**: p. 105-117. [48] | Inadequate intervention |
| Jiwani, R., et al., *Changes in Patient-Reported Outcome Measures With a Technology-Supported Behavioral Lifestyle Intervention Among Patients With Type 2 Diabetes: Pilot Randomized Controlled Clinical Trial.* JMIR Diabetes, 2020. **5**(3): p. e19268. [49] | Inadequate intervention |
| Lazo-Porras, M., et al., *Foot thermometry with mHeath-based supplementation to prevent diabetic foot ulcers: A randomized controlled trial.* Wellcome Open Res, 2020. **5**: p. 23. [50] | Inadequate intervention |
| Li, J., et al., *Cost-Effectiveness Analysis of a Mobile-Based Intervention for Patients with Type 2 Diabetes Mellitus.* Int J Endocrinol, 2021. **2021**: p. 8827629. [51] | Inadequate intervention |
| Lukkahatai, N., et al., *Feasibility of Using Mobile Technology to Improve Physical Activity Among People Living with Diabetes in Asia.* Asian Pac Isl Nurs J, 2021. **5**(4): p. 236-247. [52] | Inadequate intervention |
| Martin, M., et al., *The Influence of Baseline Hemoglobin A1c on Digital Health Coaching Outcomes in Adults With Type 2 Diabetes: Real-World Retrospective Cohort Study.* JMIR Diabetes, 2021. **6**(2): p. e24981. [53] | Inadequate intervention |
| Pelletier, C., et al., *Using an activity tracker to increase motivation for physical activity in patients with type 2 diabetes in primary care: a randomized pilot trial.* Mhealth, 2021. **7**: p. 59. [54] | Inadequate intervention |
| Sittig, S., et al., *Incorporating Behavioral Trigger Messages Into a Mobile Health App for Chronic Disease Management: Randomized Clinical Feasibility Trial in Diabetes.* JMIR Mhealth Uhealth, 2020. **8**(3): p. e15927. [55] | Inadequate intervention |
| Tu, Y.Z., et al., *The Effects of Continuous Usage of a Diabetes Management App on Glycemic Control in Real-world Clinical Practice: Retrospective Analysis.* J Med Internet Res, 2021. **23**(7): p. e23227. [56] | Inadequate intervention |
| Whittemore, R., et al., *¡Sí, Yo Puedo Vivir Sano con Diabetes! A Self-Management Randomized Controlled Pilot Trial for Low-Income Adults with Type 2 Diabetes in Mexico City.* Curr Dev Nutr, 2020. **4**(5): p. nzaa074. [57] | Inadequate intervention |
| Yasmin, F., et al., *The influence of mobile phone-based health reminders on patient adherence to medications and healthy lifestyle recommendations for effective management of diabetes type 2: a randomized control trial in Dhaka, Bangladesh.* BMC Health Serv Res, 2020. **20**(1): p. 520. [58] | Inadequate intervention |
| Ruiz, J.G., et al., *Computer-Based Programmed Instruction Did Not Improve the Knowledge Retention of Medication Instructions of Individuals With Type 2 Diabetes Mellitus.* The Diabetes Educator, 2013. **40**(1): p. 77-88. [59] | Inadequate intervention |
| Zeidi, I.M., H. Morshedi, and H. Alizadeh Otaghvar, *A theory of planned behavior-enhanced intervention to promote health literacy and self-care behaviors of type 2 diabetic patients.* J Prev Med Hyg, 2020. **61**(4): p. E601-e613. [60] | Inadequate intervention |
| Bai, L.L., et al., *USING GUESSING GAME TO INCREASE COMPLICATION AWARENESS OF PATIENTS WITH NEWLY DIAGNOSED TYPE 2 DIABETES.* Acta Endocrinol (Buchar), 2018. **14**(3): p. 401-407. [61] | Inadequate intervention |
| del Pozo-Cruz, J., et al., *A Primary Care–Based Randomized Controlled Trial of 12-Week Whole-Body Vibration for Balance Improvement in Type 2 Diabetes Mellitus.* Archives of Physical Medicine and Rehabilitation, 2013. **94**(11): p. 2112-2118. [62] | Inadequate intervention |
| Wieland, M.L., et al., *Pilot Feasibility Study of a Digital Storytelling Intervention for Immigrant and Refugee Adults With Diabetes.* The Diabetes Educator, 2017. **43**(4): p. 349-359. [63] | Inadequate intervention |
| Dehghani Zahedani, A., et al., *Improvement in Glucose Regulation Using a Digital Tracker and Continuous Glucose Monitoring in Healthy Adults and Those with Type 2 Diabetes.* Diabetes Ther, 2021. **12**(7): p. 1871-1886. [64] | Inadequate study design |
| Ruggiero, L., et al., *Diabetes Island: Preliminary Impact of a Virtual World Self-Care Educational Intervention for African Americans With Type 2 Diabetes.* JMIR Serious Games, 2014. **2**(2): p. e10. [65] | Inadequate study design |
| Senior, H., et al., *Investigating innovative means of prompting activity uptake in older adults with type 2 diabetes: a feasibility study of exergaming.* J Sports Med Phys Fitness, 2016. **56**(10): p. 1221-1225. [66] | Inadequate study design |
| Wood, F.G., et al., *Interactive Multimedia Tailored to Improve Diabetes Self-Management.* Nursing Clinics, 2015. **50**(3): p. 565-576. [67] | Inadequate study design |
| Mounié, M., et al., *Cost-Effectiveness Evaluation of a Remote Monitoring Programme Including Lifestyle Education Software in Type 2 Diabetes: Results of the Educ@dom Study.* Diabetes Ther, 2022. **13**(4): p. 693-708. [68] | Inadequate study design |
| Debong, F., H. Mayer, and J. Kober, *Real-World Assessments of mySugr Mobile Health App.* Diabetes Technol Ther, 2019. **21**(S2): p. S235-s240. [69] | Inadequate study design |
| Álvarez-Barbosa, F., et al., *Is the Wii balance board a valid and reliable instrument to assess postural stability in older adults with type 2 diabetes mellitus?* Diabetes Res Clin Pract, 2020. **166**: p. 108313. [70] | Inadequate study design |
| Anderson, D., et al., *International feasibility study for the Women's Wellness with Type 2 Diabetes Programme (WWDP): An eHealth enabled 12-week intervention programme for midlife women with type 2 diabetes.* Diabetes Res Clin Pract, 2021. **171**: p. 108541. [44] | Inadequate intervention |
| Cuevas, H. and S. Carter, *Online Cognitive Training: An Adaptation of the Memory, Attention, and Problem Solving Skills for Persons With Diabetes Intervention.* Comput Inform Nurs, 2020. **39**(3): p. 162-169. [71] | Inadequate study design |
| Timurtas, E., et al., *Technology-based and supervised exercise interventions for individuals with type 2 diabetes: Randomized controlled trial.* Prim Care Diabetes, 2022. **16**(1): p. 49-56. [45] | Inadequate intervention |
