# Supplementary figures and images for: Effectiveness of Digital Health Interventions Containing Game Components for the Self-management of Type 2 Diabetes: Systematic Review
Source: JMIR Serious Games. 2023 Jun 1;11:e44132. doi: 10.2196/44132 (PMC10273035; doi:10.2196/44132)

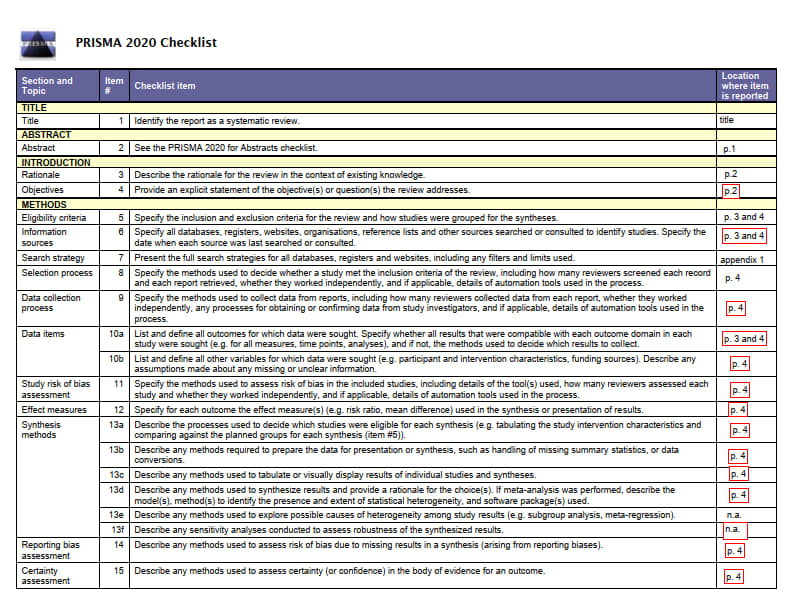

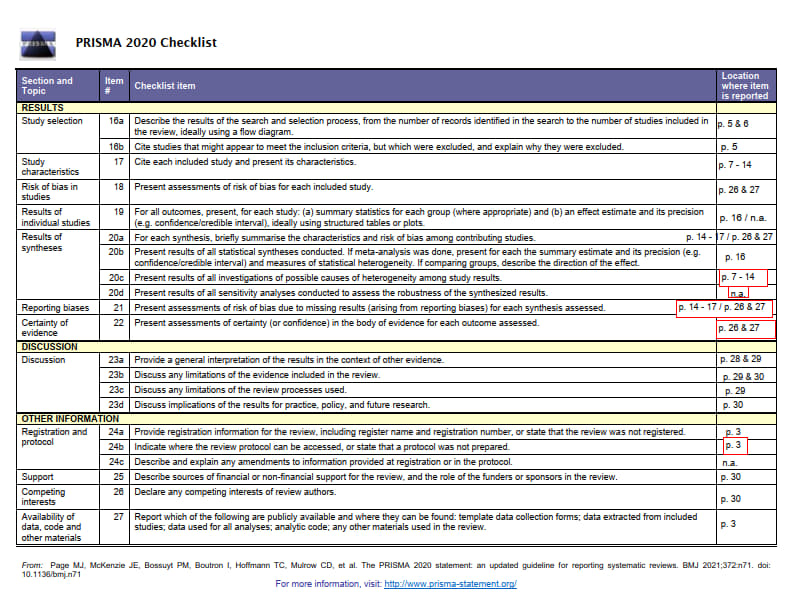

Supplement: Multimedia Appendix 3 [file games_v11i1e44132_app3.docx]
